# ANIMAL MODELS FOR COLORECTAL CANCER

**DOI:** 10.1590/0102-672020180001e1369

**Published:** 2018-07-02

**Authors:** Alana Serrano Campelo DE-SOUZA, Thais Andrade COSTA-CASAGRANDE

**Affiliations:** 1 Mestrado Profissional em Biotecnologia Industrial, Universidade Positivo, Curitiba, Paraná,Brazil

**Keywords:** Models, animal, Colorectal neoplasms, Carcinogens., Modelos animais, Neoplasias colorretais, Carcinógenos.

## Abstract

***Introduction*:**

Colorectal cancer is a very frequent sort of neoplasm among the population, with a high mortality rate. It develops from an association of genetic and environmental factors, and it is related to multiple cell signaling pathways. Cell cultures and animal models are used in research to reproduce the process of disease development in humans. Of the existing animal models, the most commonly used are animals with tumors induced by chemical agents and genetically modified animals.

***Objective*:**

To present and synthesize the main animal models of colorectal carcinogenesis used in the research, comparing its advantages and disadvantages.

***Method*:**

This literature review was performed through the search for scientific articles over the last 18 years in PubMed and Science Direct databases, by using keywords such as “animal models”, “colorectal carcinogenesis” and “tumor induction”.

***Results*:**

1,2-dimethylhydrazine and azoxymethane are carcinogenic agents with high specificity for the small and large intestine regions. Therefore, the two substances are widely used. Concerning the genetically modified animal models, there is a larger number of studies concerning mutations of the *APC*, *p53* and *K-ras* genes. Animals with the *APC* gene mutation develop colorectal neoplasms, whereas animals with *p53* and *K-ras* genes mutations are able to potentiate the effects of the *APC* gene mutation as well as the chemical inducers.

***Conclusion*:**

Each animal model has advantages and disadvantages, and some are individually efficient as to the induction of carcinogenesis, and in other cases the association of two forms of induction is the best way to obtain representative results of carcinogenesis in humans.

## RESUMO

***Introdução*:**

O câncer de cólon e reto é bastante frequente na população e com elevado índice de mortalidade. Ele se desenvolve a partir da associação de fatores genéticos e ambientais e está relacionado a múltiplas vias de sinalização celular. Para o estudo da doença são utilizados cultivos celulares e modelos animais, que sejam capazes de reproduzir o processo de desenvolvimento da doença em humanos. Dos modelos existentes, os mais comumente utilizados são os animais induzidos ao desenvolvimento tumoral por agentes químicos e os animais geneticamente modificados.

***Objetivo*:**

Apresentar e sintetizar os principais modelos animais de carcinogênese colorretal utilizados na pesquisa, comparando suas vantagens e desvantagens.

***Método*:**

Para o desenvolvimento dessa revisão foi realizada uma busca por artigos científicos dos últimos 18 anos nas bases de dados PubMed e Science Direct, utilizando como palavras-chave “modelos animais”, “carcinogênese colorretal” e “indução tumoral”.

***Resultado*:**

O 1,2 dimetilhidrazina e o azoximetano são agentes carcinógenos com alta especificidade para o intestino delgado e grosso. Por isso, as duas substâncias são amplamente utilizadas. Dos modelos animais geneticamente modificados observa-se maior quantidade de estudos referentes às mutações dos genes *APC, p53*e*K-ras*. Os animais com mutação do gene *APC* desenvolvem neoplasias colorretais, enquanto que animais com mutações dos genes *p53* e *K-ras* são capazes de potencializar os efeitos da mutação do gene *APC*, bem como dos indutores químicos.

***Conclusão*:**

Cada modelo animal apresenta vantagens e desvantagens, sendo que alguns são individualmente eficientes na indução da carcinogênese, e em outros casos a associação de duas formas de indução é a melhor maneira de se obter resultados representativos da carcinogênese em humanos.

## INTRODUCTION

Cancer is a sort of disease that can reach most of the organs and tissues of the human body. It is characterized primarily by the disordered growth of cells, often able to metastasize to other regions of the body. According to Petit et al.^19^, the disease is associated with both genetic factors, inherent to each individual, and environmental factors.

Colorectal cancer (CRC) is the 4^th^ most frequent neoplasm among the world’s population, accounting for 694,000 of the 8.2 million cancer deaths in 2012^26^. It is a very aggressive type of cancer that has a high potential to spread to other organs. It develops in 90-95% of cases due to environmental factors^2,14^ and it is related to multiple signal transduction cascades, important for different types of biological response such as angiogenesis, apoptosis and cell proliferation^24^ ^,^ ^29^.

CRC may be hereditary or sporadic, accounting for 80% of all patients affected by the disease. The hereditary form is related to two familial syndromes, familial adenomatous polyposis, in which the appearance of multiple intestinal polyps, and hereditary nonpolyposis colorectal cancer is observed. Individuals who develop familial adenomatous polyposis have a mutation in the *APC* (adenomatous polyposis of the colon) tumor suppressor gene, whereas those who develop hereditary nonpolyposis colorectal cancer have mutations in genes involved in DNA repair and mismatch repair (MMR) genes^6^. The sporadic form, in turn, is related to inflammatory bowel processes such as Crohn’s disease and ulcerative colitis, as well as to eating habits such as red meat consumption and low fiber intake^12^ ^,^ ^19^.

For studies related to the development, treatment and prevention of colon and rectum tumors, animal models or cell cultures, which are representative of a carcinogenic situation in humans, are used. However, in spite of all the ethical conflicts involved in animal experimentation^20^, one of the major disadvantages of cell culture, compared to animal models, is the inability to reproduce metastatic and angiogenesis situations^11^.

For research purposes, the two major animal models of colorectal carcinogenesis are colorectal tumors induced by chemical or environmental agents in rodents, which represent sporadic CRC, and genetically modified mice, which represent the hereditary familial adenomatous polyposis and hereditary non-polyposis colorectal cancer syndromes^11^.

This review aims to present and synthesize the main animal models of colorectal carcinogenesis used in the research, comparing its advantages and disadvantages.

## METHOD

For the development of this review, a search for scientific articles over the last 18 years in the PubMed and Science Direct databases was carried out, using keywords such as “animal models”, “colorectal neoplasias” and “induced colon cancer”. The search resulted in 89 articles, of which 31 were selected as the most relevant for this review. 

## RESULTS

### Colorectal cancer induced by exogenous agents

According to Andrade et al.^3^ there are several factors capable of increasing the chances of developing colorectal cancer, among them the intake of foods rich in fat, and with low fiber content. Thus, the eating habits of an individual can directly influence the occurrence of this neoplasia^3^.Newmark et al.^17^ observed that 25% of the animals fed for two years on this diet had invasive adenocarcinomas in small bowel, cecum and proximal colon regions; and that another group, fed with a diet enriched with calcium and vitamin D, did not present intestinal lesions. In addition, Yang et al.^28^ observed that 75% of the animals, treated for a year and a half with the same lipid diet, had the *APC-/*+ mutation and 57% the *Muc2-/*- mutation, which are important in CRC development. However, only 27% of them had alterations in the intestinal mucosa.The two studies showed that diet can directly influence the appearance of colorectal neoplasias, as it promotes reprogramming of intestinal cells, thus representing the spontaneous colorectal cancer model in humans. However, although the authors observed different intestinal neoformations in the animals treated with the hyper caloric diet, it was not possible to evaluate which mutations were responsible for the development of the tumors. Also, the diet may not be considered the best model of colorectal carcinogenesis, since the percentage of animals that develop the neoplasia is small, and the time for this to occur is long.

 Besides the diet, chemical substances with carcinogenic potential are also used. The two most commonly used tumor inducers in animal models for induction of sporadic CRC are azoxymethane (AOM), which is a direct inducer, and 1,2 dimethylhydrazine (DMH), which is an indirect inducer of carcinogenesis. They are able to represent the mechanisms of development of CRC that occur naturally in humans, being very useful models in studies that aim to study chemopreventive and chemotherapeutic effects of other substances^18^.

DMH is a pro-carcinogenic agent for colon cancer, it is activated in the liver and transported to the intestine by bile and blood. Its use promotes the production of free radicals, which are responsible for causing oxidative damage to the DNA of colon and liver cells^23^.

According to Burlamaqui et al.^4^, AOM is an active metabolite of DMH also used for tumor induction in rodents. It mainly affects organs such as the liver, the lungs and the colon, and the lesions found are directly proportional to time of exposure and the dose administered^13^.

In 2014, Lahouar et al.^13^ using AOM as an inducer, observed that animals in the AOM group had significantly more aberrant crypts, which are pre-neoplastic lesions, than the animals in the control group. In addition, they also noticed the appearance of inflammatory lesions and histological changes in the hepatic and pulmonary tissues of the animals. By 2015, Yu et al.^31^, in addition to observing pre-neoplastic lesions in mice treated with AOM, also observed a 51% and 46% increase in pro-inflammatory cytokines, such as tumor necrosis factor alpha (TNF-a) and interleukin 6 (IL-6), respectively.

Aachary et al.^1^ observed that animals treated with DMH developed outbreaks of aberrant crypts and also presented alterations in the intestinal microbiota. When compared to the control group, they showed a reduction in the number of *Bifidobacteria* and an increase in *E. coli* and *C. perfringens* species.

Umesalma and Sudhandiran^25^ demonstrated that animals treated with DMH showed an increased expression of inflammatory markers cycloxigenase 2 (cox-2) and IL-6. The control group presented 0.6% and 1.4% of cells expressing the two markers, respectively, and the DMH group presented 1.2% and 3.5% expressions.

DMH was also effective in CRC induction in a study by Youssef et al.^30^ which detected about 100.5 aberrant crypts foci in the 10 animals that received the inducer, and a total of 20 colorectal tumors in these same individuals, whereas the animals in the control group had neither pre-neoplastic lesions nor neoformations.

Both substances are used for CRC induction in rodents. However, there is controversy over which of the two is the most effective in tumor induction. Burlamaqui et al.^4^ stated that AOM is a more potent inducer than DMH because it is activated faster in the body. However, in a comparative study done by Jucá et al.^12^ it was observed that the induction by DMH promoted the appearance of dysplasias in mild, moderate and severe degree, in addition to carcinomas in situ, whereas the induction by AOM promoted only moderate dysplasia in the colon of the animals.

Since AOM is an active metabolite of DMH, the mechanism of induction of carcinogenesis promoted by the two substances is very similar. In the studies mentioned, the formation of aberrant crypts foci and intestinal inflammatory processes was observed in the animals treated with both DMH and AOM. However, DMH seems to be a more effective agent because in addition to the pre-neoplastic and inflammatory lesions, it also promoted the development of intestinal adenomas and carcinomas. Thus, from the models induced by exogenous agents it can be concluded that all agents, diet, AOM and DMH are able to induce CRC. But the diet proves to be a slow and low-efficiency model; the AOM is an inducer of mainly aberrant crypts foci and inflammation, and DMH is an inducer of colorectal tumors in more advanced stages, showing to be the most efficient model.

### Genetically modified animals

Many genes are involved in colorectal carcinogenesis, including tumor suppressors *APC, DCC* (deleted in colorectal cancer), *p53* (gene encoding tumor protein p53) and *MCC* (mutated in colorectal cancer), the oncogenes *K-ras*(Kirsten rat sarcoma viral oncogene homolog), *SRC*(proto-oncogene SRC) and *C-myc* (homologous oncogene viral myelocytomatosis), the DNA repair genes *hMSH2* (mutS 2 homologue), *hMSH6* (mutS 6 homologue), *hMLH1* (mutL 1 homologue), *hPMS1* (BstNIproline rich protein subfamily 1) and *hPMS2* (BstN1 proline rich protein subfamily 2), in addition to *CD44* genes (gene encoding CD44 molecule) and *COX-2* (cytochrome C oxidase, subunit 2). Each of them acts differently for the development of colorectal neoplasms, and mutations in two or more of these genes are often related to the malignancy profile of the tumors^6^.

Thus, there are innumerable genetically modified animal models that have been developed from the knowledge about the genetic factors involved in the development of the disease^6^. However, only the models whose mutations are most frequent in sporadic CRC in humans (*APC*> 70%, *p53*> 60% and *K-ras*> 40%) and in hereditary familial adenomatous polyposis*(APC)* and hereditary nonpolyposis colorectal cancer*(MMR)* ^11^ will be described next.

*APC* ^min^ animals are genetically modified that have a mutation in the *APC* gene. The acronym “min” means multiple intestinal neoplasms, and this is an autosomal dominant mutation, which in homozygous conditions is lethal to animals. Animals that are heterozygous for the mutation develop important anemic conditions at 60 days of life and develop tumors in the large and small intestine. As in familial adenomatous polyposis cases, *APC* ^min^ animals also develop colorectal adenomas, but they die at 120 days of life^27^.

Knockout animals for *p53* gene rarely develop colorectal tumors. Reed et al.^21^ reported in 2008 that *p53* knockout animals did not develop colorectal neoplasias; however, the association of *APC* ^min^ and *p53* knockout mutations promoted an increase in aberrant crypts foci number when compared to *APC* ^min^ animals. In addition, in 2008 Hu et al.^10^ reported that an association of *p53* knockout animals with tumor inducer AOM was efficient in inducing carcinogenesis in the colon of the animals, and also to potentiate the action of the AOM. The same could be observed by Sakai et al.^22^ in which *p53* knockout animals showed only neoplastic development when placed in contact with the DMH inducer.

In addition to animals with mutations in the *APC*and *p53*genes, mice with mutations in codon 12 of the *K-ras* gene are also used. From a homologous recombination process, one of the *K-ras* gene alleles is replaced by a mutated *K-ras* ^G12D^ allele in which there is the substitution of a guanine with an adenine at the second base of codon 8. According to Haigis et al.^9^ animals with this mutation have regions of hyperplasia in the colon as well as aberrant crypt and ring cells. Associating the *K-ras* ^G12D^ and *APC* ^min^ mutations, Calcagno et al.^5^ and Luo et al^16^ reported an increase in the number of lesions in the colon, as well as the presence of completely undifferentiated cells.

Another mutation found in colorectal tumors is the *K-ras* ^v12^ mutation, in which the substitution of a guanine for a thymine occurs. In 2007, Luo et al.^15^ demonstrated that the *K-ras* ^v12^ mutation alone is not capable of inducing tumorigenesis, but once it is associated with mutations in repair genes, such as the *MSH2* gene, it promotes and accelerates tumor development. *K-ras* ^v12^/*Msh2-*/- animals presented a greater number of tumors, both in the small and in the large intestine, than *Msh2-/-* animals: ranging from 1.41 to 7.75 tumors per mouse in the small intestine and from 0.13 to 2.7 tumors per rat in the large intestine.

These mutations in DNA repair genes are representative of hereditary nonpolyposis colorectal cancer. According to Edelmann et al.^7^ 7/22 (32%) mice *MLH1+/-* and 13/18 (72%) of *MLH1-/-* mice developed tumors, against 1/20 (5%) mice without the mutation. In addition, they also observed that the survival of animals was lower in *MLH1-/-* animals than in *MLH1+/-*, 7 and 9.8 months, respectively,

Among the genetically modified models, the one that best represents the hereditary CRC are the *APC* ^min^ animals, among which the formation of intestinal tumors is observed. The other mutants only become representative models when an association of two or more types of mutation is made, or through the association of a mutation with induction by an exogenous agent, such as diet, for example. All models presented, both induced by exogenous agents and those genetically modified, have advantages and disadvantages (Table 1) for the study of colorectal carcinogenesis. However, some of them, such as tumor induction by DMH and *APC* ^min^ animals, are more suitable for research because of their ability to develop colorectal neoplasias, without the need to associate two or more induction methods.

**TABLE 1:** Animal models used for research in colorectal cancer, and their main advantages and disadvantages

Animal Model	CRC type	Advantages	Disadvantages	References
Western Diet	Sporadic	Induction of carcinogenesis in small intestine, cecum and proximal colon.	Dietary mutations have not been described yet. Few animals develop neoplastic lesions and the time of development is rather long.	17, 28
DMH	Sporadic	Capacity to induce metastasis. Induction of adenomas and adenocarcinomas. High degree of specificity for intestine.	Indirect inducer. Promotes liver toxicity.	23
AOM	Sporadic	Direct inducer. High degree of specificity for intestine.	More expensive than DMH. Promotes liver toxicity.	4
APC	SporadicandHereditary (PAF)	Good for study of hereditary CRC.	Animals die within 120 days.	27
P53	Sporadic	It potentiates the action of other genes or tumor inducers.	Inhibition of gene expression alone was not able to induce colorectal carcinogenesis.	21
K-ras	Sporadic	Hyperplasia and presence of aberrant crypts. It potentiates tumorigenesis of other mutations.	They do not develop tumors, only pre-neoplastic lesions. Association with other mutations and/or inducers is required.	9, 15
MMR	Hereditary (HNPCC)	Good for study of hereditary CRC (HNPCC).	Most tumors are in the small intestine.	7, 15

## DISCUSSION

 Animal models are important for studies of the development and pathogenesis of colorectal tumors, as well as for the evaluation of possible risk factors, preventive agents and treatments. As shown, some individual models are representative of CRC in humans and considered to be good animal models for this type of study. However, it is noted that the association of at least two methods of colorectal carcinogenesis induction is required for most models. This is due to the fact that colon cancer is a multifactorial disease that develops by the presence of multiple factors, both genetic and environmental.

## CONCLUSION

Each animal model has advantages and disadvantages, and some models are individually efficient as to the induction of carcinogenesis, and in other cases the association of two forms of induction is the best way to obtain representative results of carcinogenesis in humans.
